# Polyphenolic Compounds Activate SERCA1a and Attenuate Methylglyoxal- and Palmitate-Induced Impairment in Pancreatic INS-1E Beta Cells

**DOI:** 10.3390/cells13221860

**Published:** 2024-11-09

**Authors:** Vladimir Heger, Barbora Benesova, Magdalena Majekova, Petronela Rezbarikova, Attila Hunyadi, Lubica Horakova, Jana Viskupicova

**Affiliations:** 1 Institute of Experimental Pharmacology and Toxicology, Centre of Experimental Medicine of the Slovak Academy of Sciences, 841 04 Bratislava, Slovakia; vladimir.heger@medirexgroup.sk (V.H.); barbora.benesova@savba.sk (B.B.); magdalena.majekova@savba.sk (M.M.); petronela.zizkova@savba.sk (P.R.); exfahorl@savba.sk (L.H.); 2 Faculty of Natural Sciences, Comenius University, 841 04 Bratislava, Slovakia; 3 Institute of Pharmacognosy, University of Szeged, H-6720 Szeged, Hungary; hunyadi.attila@szte.hu

**Keywords:** activators, insulin release, pancreatic beta cells, polyphenols, SERCA

## Abstract

Sarco/endoplasmic reticulum Ca^2+^-ATPase (SERCA) is an important regulatory protein responsible for maintaining calcium homeostasis within cells. Impairment of SERCA associated with activity/expression decrease has been implicated in multiple chronic conditions, including cardiovascular diseases, diabetes, cancer, neurodegenerative diseases, and skeletal muscle pathologies. Natural polyphenols have been recognized to interact with several target proteins involving SERCA. To date, only a limited number of polyphenolic compounds or their derivatives have been described either to increase SERCA activity/expression directly or to affect Ca^2+^ signaling pathways. In this study, we tested polyphenols for their ability to activate SERCA1a in the absence or presence of methylglyoxal or palmitate and to impact insulin release in pancreatic beta cells. The protective effects of these compounds against methylglyoxal- or palmitate-induced injury were evaluated. Results indicate that 6-gingerol, resveratrol, and ellagic acid activate SERCA1a and protect against activity decrease induced by methylglyoxal and palmitate. Molecular docking analysis revealed the binding of these polyphenols to Glu439 in the SERCA1a P-domain, suggesting a critical role in the stimulation of enzyme activity. Ellagic acid was found to directly stimulate the activity of SERCA1a, marking the first instance of such an observation.

## 1. Introduction

Sarco/endoplasmic reticulum Ca^2+^-ATPases (SERCAs) belong to the most studied membrane transporters. SERCA is responsible for maintaining low intracellular calcium levels (~10–100 nmol/L) by transporting Ca^2+^ ions from the cytosol into the SR/ER using energy derived from ATP hydrolysis. Its correct functioning is inevitable for muscle contraction/relaxation, cell signaling, cellular energetics, as well as maintenance of Ca^2+^ homeostasis. SERCAs are encoded by three genes generating several tissue-specific SERCA isoforms (SERCA1a, b; SERCA2a-d; SERCA3a-f) [1,2]. Impairment in their regulation has been implicated in various pathophysiological conditions, such as cardiomyopathy, heart failure, vascular complications, diabetes, cancer, dystrophy, neurodegenerative diseases, and Brody’s and Darier’s disease [3]. Pancreatic beta cells express three isoforms (SERCA2a, SERCA2b, SERCA3), with SERCA2b prevailing as the predominant isoform, thus serving as the primary regulator of ER Ca^2+^ transport in these cells [4]. Increasing evidence indicates that diminished SERCA2b function is a critical factor that contributes to the initiation of endoplasmic reticulum (ER) stress, thus triggering a complex signaling cascade known as the unfolded protein response [5]. Downregulated levels of SERCA2b were observed in INS-1E cells after exposure to high glucose concentrations in vitro [6,7] as well as in experimental models of diabetes [8], resulting in diminished insulin secretion and Ca^2+^ homeostasis impairment. Overexpression of SERCA2a by gene therapy has proven successful in clinical models of heart failure, demonstrating its potential therapeutic efficacy [9]. Combining bone marrow mesenchymal stem cells and the SERCA1a gene has shown remarkable effectiveness in treating muscle degeneration caused by diabetes, indicating a potential for targeting SERCA1a in diabetic muscle therapies [10]. Hence, targeting SERCAs from different tissues to regulate Ca^2+^ homeostasis may represent a promising therapeutic strategy for various disease states, including diabetes.

In the context of diabetes, chronic exposure to high glucose levels and its metabolites (such as methylglyoxal), as well as elevated levels of free fatty acids, primarily palmitate, has been linked to disruption of Ca^2+^ homeostasis. The interplay of glucotoxicity and lipotoxicity contributes to Ca^2+^ dysregulation through the generation of oxidative and ER stress, mitochondrial dysfunction, and eventual beta cell death [11]. Therefore, targeting calcium dysregulation, ER, and oxidative stress could be a promising strategy for treating metabolic diseases associated with glucolipotoxicity.

Polyphenols belong to a diverse group of secondary plant metabolites with an extensive array of bioactivities targeting different cellular pathways and molecular mechanisms. Due to their potent antioxidant and anti-inflammatory properties, they serve as multitarget compounds effective against inflammation and oxidative stress in metabolic conditions [12]. Clinical trials suggest that consuming polyphenols may lower blood glucose levels in individuals with type 2 diabetes or those at risk and may enhance the effects of anti-diabetic drugs [13]. Moreover, polyphenols are promising antidiabetic candidates with their ability to inhibit amyloid formation [14]. These phytochemicals are known to modulate both cellular signaling and gene expression, thereby intervening in diverse intracellular processes [15]. The direct binding of these compounds to target proteins, including SERCAs, has been described as an effective health-promoting mechanism underlying polyphenol-mediated protective action [16]. In our recent review article, we highlighted the capacity of specific natural polyphenols to modulate the activity/expression of distinct SERCA isoforms or influence Ca^2+^-signaling cascades, contributing to the amelioration of various chronic pathological conditions [3]. Increasing evidence suggests that polyphenols may have the potential to counteract the adverse effects of lipotoxicity and glucotoxicity, thereby preserving, restoring, and enhancing the normal functions of beta cells [17,18,19].

Current knowledge provides limited insights into how specific activators influence SERCA pumps and related processes in calcium signaling and metabolic diseases. Therefore, the objective of this study was to explore the therapeutic potential of pharmacological SERCA pump activation by natural polyphenols. The protective effects of these compounds were assessed under conditions simulating diabetes, specifically, in the presence of methylglyoxal (MGX) and palmitate (PAL). The evaluation was conducted at both the protein level of the isolated SERCA1a protein and the cellular level utilizing pancreatic beta cells (INS-1E cell line). The research aimed to address the following key questions:

1. Can the polyphenolic compounds in the data set directly stimulate the activity of the SERCA1a isoform in the non-cellular system?
2. What impact do polyphenols have on the viability of the pancreatic INS-1E beta cell line? Are there any correlations among SERCA activity, beta cell viability, and insulin secretion?
3. Are the selected compounds capable of protecting SERCA1a and pancreatic beta cells against impairments induced by MGX and PAL?
4. What molecular mechanisms may underlie SERCA1a activation based on a molecular modeling study?

This study conducts a preliminary screening to investigate the activation of SERCA1a by polyphenols, addressing the limited knowledge on the impact of diabetes on skeletal muscle homeostasis. The study also aims to assess the effectiveness of polyphenols in protecting against damage caused by the most common diabetes-related compounds, MGX and PAL.

## 2. Materials and Methods

### 2.1. Polyphenolic Compounds

Resveratrol was purchased from Career Henan Chemical Co. (Zhengzhou, China) and had > 98% purity. Oxyresveratrol, [6]-gingerol, and [6]-shogaol were synthesized previously and were available in >95% purity using HPLC-PDA. Briefly, oxyresveratrol was isolated from *Morus nigra* roots by using a multistep chromatographic purification technique as published before [20]. [6]-Gingerol was purified from a commercial ginger extract purchased from Xi’an Pincredit Bio-Tech Co., Ltd. (Xi’an, China) by flash chromatography using a CombiFlash Rf+ Lumen instrument equipped with an integrated evaporative light-scattering detector (Teledyne Isco, Lincoln, NE, USA), a RediSep Gold silica column, and a gradient elution of acetone (from 0% to 15%) in n-hexane. An aliquot of 4 g of extract was separated, and a 36.8% yield of [6]-gingerol was obtained, as previously published [21]. [6]-Shogaol was subsequently semi-synthesized from [6]-gingerol using the method reported by Wei et al. [22] with slight modifications, as published before [21]. Briefly, p-toluenesulphonic acid was used as a dehydrating agent and toluene as a solvent under reflux for 15 min. The product was purified by solid-phase extraction over silica using n-hexane–acetone (8:2, v/v) followed by flash chromatography on the instrument as mentioned above using a gradient elution of 0–5% of acetone in n-hexane and achieving a yield of 46.5%. Other polyphenols of analytical purity were purchased from Sigma (St. Louis, MO, USA).

### 2.2. Sarcoplasmic Reticulum Vesicle Isolation

Sarcoplasmic reticulum (SR) vesicles were isolated from the fast-twitch skeletal muscle of a New Zealand female rabbit (approximately 2.5 kg) as previously described [23,24,25]. The fast-twitch skeletal muscle was obtained post-mortem at the Research Institute for Animal Production Nitra of the National Agricultural and Food Center. The tissue was collected according to the institute’s standard protocols, which adhere to national animal welfare regulations. Briefly, 100 g of rabbit skeletal muscle was thoroughly mixed with 150 mL of buffer (pH 8.0) containing 0.3 mol/L sucrose, 20 mmol/L L-histidine, 1 mmol/L dithiotreitol (DTT), and 5 μmol/L phenylmethylsulfonyl fluoride (PMSF) in 96% ethanol at 2–4 °C. The homogenate was centrifuged for 15 min at 8000× *g* at 4 °C. The supernatant was filtered and centrifuged for 90 min at 37,000× *g* at 4 °C. The resulting sediment was homogenized in 30 mL of buffer (pH 8.0) containing 0.3 mol/L sucrose, 10 mmol/L L-histidine, 0.6 mol/L KCl, 1 mmol/L DTT, and 5 μmol/L PMSF in 96% ethanol at 2–4 °C using a piston homogenizer. Subsequently, it was centrifuged for 2 h at 37,000× *g* at 4 °C. The sediment containing SR vesicles was resuspended in buffer (pH 8.0) containing 0.25 mol/L sucrose, 1 mol/L KCl, 50 mmol/L K_2_HPO_4_, and 50 mmol/L KH_2_PO_4_ and dialyzed overnight in the same buffer at 2–4 °C. Finally, the SR vesicles were aliquoted and stored at −80 °C. Protein content was assessed by the Lowry assay using bovine serum albumin as a standard (Bio-Rad Laboratories, Richmond, CA, USA).

### 2.3. Treatment of SR Vesicles with MGX, PAL, and Polyphenols

Sarcoplasmic reticulum vesicles were treated with individual polyphenols (5–200 μM) for 2 min with or without MGX (3 mM) and PAL (0.65 mM) before activity measurement. PAL was dissolved in 50% ethanol to create a stock solution with a concentration of 100 mM. This stock solution was then added to a pre-warmed 10% w/w BSA solution at 37 °C to achieve a final concentration of 10 mM. The solution was incubated in a water bath for an additional 10 min to ensure proper complexation of PAL with BSA. For each compound, a 10 mM stock solution in DMSO was prepared, aliquoted, and stored at −20 °C.

### 2.4. SERCA1a Activity Measurement

SERCA1a activity was spectrophotometrically measured by the NADH-coupled enzyme assay following the protocol by Ortiz et al. [26] with modifications by our team. An enzyme-coupled NADH-linked ATPase assay was employed to assess SERCA1a ATPase activity in 96-well microplates, with a final amount of 1.25 μg of proteins per well. Each well contained an assay mix composed of 50 mM MOPS (pH 7.0), 100 mM KCl, 5 mM MgCl_2_, 1 mM EGTA, 0.2 mM NADH, 1 mM phosphoenolpyruvate, 10 IU/mL of pyruvate kinase, 10 IU/mL of lactate dehydrogenase, and 1 μM of the calcium ionophore A23187 (Sigma, St. Louis, MO, USA). The total reaction mixture volume was 0.25 mL. The reaction was initiated by the addition of 10 μM CaCl_2_. The reaction rate was determined by measuring the decrease in NADH absorbance at 340 nm at 37 °C using a microplate reader (Infinite M200, Tecan, Männedorf, Switzerland).

### 2.5. Cell Culture and Treatment with Polyphenols

The INS-1E insulinoma pancreatic beta cell line (kindly provided by Prof. Claes Wollheim, University of Geneva) was cultured in RPMI 1640 (11 mM glucose, Sigma, St. Louis, MO, USA). The RPMI 1640 was supplemented with 100 U/mL penicillin, 100 μg/mL streptomycin, 2 mM L-glutamine, 1 mM Na pyruvate, 55 μM 2-mercaptoethanol, 10 mM HEPES, 1% nonessential amino acids, and 10% fetal bovine serum (pH 7.0–7.4). Cells were cultivated in a humidified incubator containing 5% CO_2_ at 37 °C. All experiments were conducted using cells from passages 40 to 70. The cells were pre-treated with various concentrations of individual compounds (5–200 μM) for 24 h with or without MGX (2.5 mM) and PAL (0.4 mM). Subsequent measurements were then performed using a cytotoxicity MTT assay and an insulin release assay.

### 2.6. 3-[4,5-Dimethylthiazol-2-yl]-2,5 Diphenyl Tetrazolium Bromide (MTT) Assay

The MTT (Sigma, St. Louis, MO, USA) reduction assay was used as an indicator of cell damage and was conducted following a standard protocol. INS-1E cells (5 × 10^4^ cells per well) were seeded into 96-microwell plates. The cells were preincubated for different periods with or without various concentrations of individual compounds for 24 h (5% CO_2_ at 37 °C). MTT was introduced to achieve a final concentration of 0.5 mg/mL in an RPMI 1640 medium. Following a 4 h incubation, the MTT medium was removed. The addition of DMSO (100 µL) was added and left to stand for 15 min to solubilize the formazan formed. The absorbance was recorded at 570 nm using a microplate reader (Infinite M200, Tecan, Switzerland).

### 2.7. Insulin Release

Insulin release in response to glucose was measured in INS-1E beta cells (passages 10–30). Briefly, after 24 h preincubation with individual compounds, beta cells were washed with a glucose-free KRBH buffer. Subsequently, cells were incubated at 37 °C for 30 min in a glucose-free KRBH buffer, followed by another 30 min incubation at 37 °C in a KRBH buffer containing either 3 mM glucose (non-stimulating concentration) or 16.7 mM glucose (insulin-stimulating concentration). The supernatants were then collected and analyzed using an RIA kit (Mercodia, Uppsala, Sweden) for insulin release, with rat insulin as the standard.

### 2.8. In Silico Study

The optimal structures of the ligands were obtained by Spartan software (Spartan’20, Version 1.1.4) using the conformer search method and the MMFF94 force field and subsequent optimization using the DFT B3LYP 6-31G* method [27]. Docking studies were performed by the Molecular Operating Environment (MOE 2020.0901) modeling program [28] using a triangle matcher and the London dG score for basic docking (limit 30 poses) and the GBVI/WSA DG score for the induced-fit refinement of the geometry (limit 5 poses). Ligands were evaluated for protonation under physiological pH conditions. Ellagic acid was treated as dianion [29].

We selected the E2P state as the reference for evaluating polyphenol binding and the modulatory role of ATP. Recent data on the rate constants of individual steps in the SERCA cycle show 6 s^−1^ for the Ca^2+^E1P → Ca^2+^E2P transition and 0.11 s^−1^ for the E2P → E2 transition [30]. Since the E439A mutation significantly increases the dephosphorylation rate [31], we focused our study on a model of the E2P state using the PDB structure 2ZBE. For comparison, the PDB structure 4XOU was used to model the E1 conformation (Ca^2+^E1.ATP state). Proteins were treated using the QuickProp protocol to correct bonds and protonation states, while the Protonate3D protocol was employed for protonation adjustments. The SiteFinder function in MOE was used to select docking pockets in the cytoplasmic domains of SERCA1a. Docking poses with the lowest final score values were selected for further analysis.

### 2.9. Statistical Analysis

Statistical analysis was performed using the Bonferroni test. Data are presented as the mean ± SD of a minimum of three or more independent measurements, with each sample measured in duplicate or triplicate. Statistical significance was defined as follows: * *p* < 0.05, ** *p* < 0.01, *** *p* < 0.001, and **** *p* < 0.0001. In the respective graphs, the symbol ‘*’ indicates a significant increase compared to the corresponding control, while the symbol ‘°’ denotes a significant decrease compared to the corresponding control. Corresponding graphs were generated using the GraphPad Prism 8 program. The data were rescaled using min–max normalization according to the following formula:

normalized = (x − min(x))/(max(x) − min(x))

## 3. Results

### 3.1. Effect of Polyphenols on SERCA1a Activity and INS-1E Cell Viability

**Figure 1:**
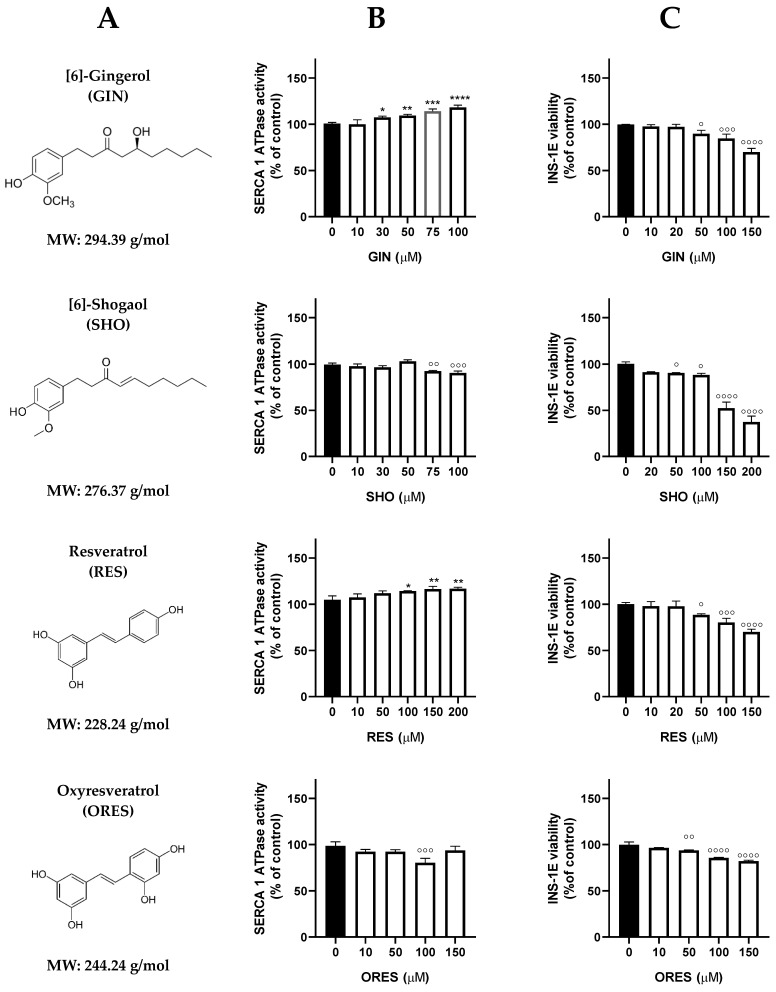

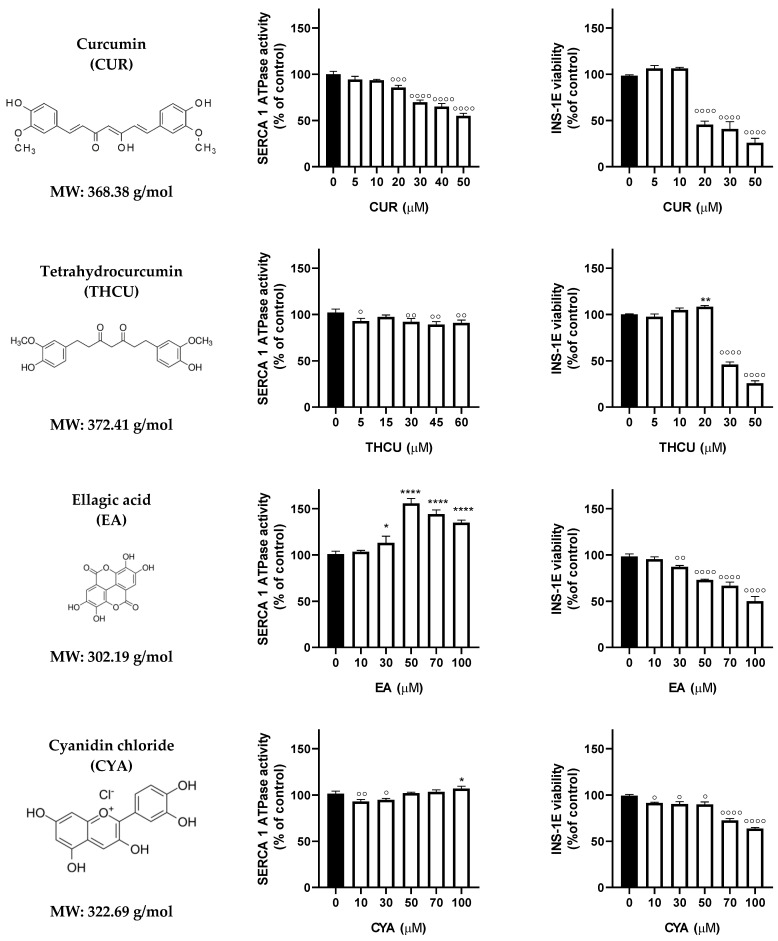

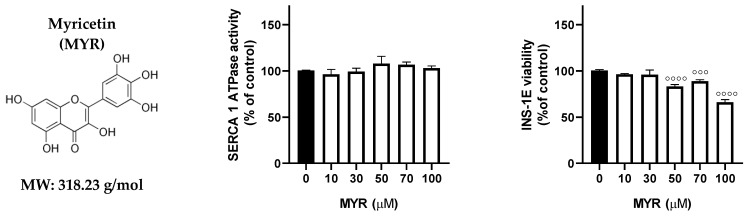
Effect of polyphenolic compounds on sarco/endoplasmic reticulum Ca^2+^-ATPase (SERCA1a) activity in the sarcoplasmic reticulum (SR) and viability of INS-1E beta cells. (**A**) Trivial names, short names, chemical structures, and molecular weights of the compounds. (**B**) SERCA1a activity measured by the NADH-coupled enzyme assay. SR vesicles (1 mg prot./mL) were incubated with polyphenolic compounds (5−200 μM) for 2 min at 37 °C, pH 7.2. (**C**) Viability of INS-1E beta cells in the presence of polyphenolic compounds. The cells (5 × 10^4^ cells/well) were preincubated for 24 h with or without varying concentrations of individual polyphenolic compounds (5−200 μM) before the MTT assay. * *p* < 0.05, ** *p* < 0.01, *** *p* < 0.001, **** *p* < 0.0001 denote a significant increase, while ° *p* < 0.05, °° *p* < 0.01, °°° *p* < 0.001, °°°° *p* < 0.0001 indicate a significant decrease in Ca^2+^-ATPase activity or beta cell viability influenced by individual compounds compared to non-treated samples.

The rationale behind the compound selection for testing lies in their reported potential to either activate or upregulate certain SERCA isoforms [3], which may subsequently offer protection to beta cells against injury induced by methylglyoxal (MGX) or palmitate (PAL). Oxyresveratrol (ORES) and a derivative of [6]-gingerol (GIN), shogaol (SHO), were chosen due to their better solubility, bioavailability, and antioxidant properties. In addition, curcumin (CUR) and tetrahydrocurcumin (THCU) were added to the data set due to their potent bioactivities, in particular antioxidant, anti-inflammatory, and antidiabetic effects [32]. Furthermore, myricetin (MYR) and cyanidin chloride (CYA), members of flavonoids, were implemented based on structural features responsible for strong biological properties to diversify the existing dataset. The screening of individual compounds tested on SERCA1a activity in sarcoplasmic vesicles as well as on the viability of pancreatic INS-1E beta cells is summarized in Figure 1. Among the polyphenols tested, GIN, RES, EA, and CYA demonstrated the ability to increase SERCA1a activity. Notably, the most potent concentration-dependent activating effect was observed with GIN and EA starting from concentrations as low as 30 µM. On the other hand, none of the substances showed a stimulating effect on cell viability except for THCU (20 µM).

### 3.2. Effect of Methylglyoxal and Palmitate on SERCA1a Activity and INS-1E Cell Viability

**Figure 2:**
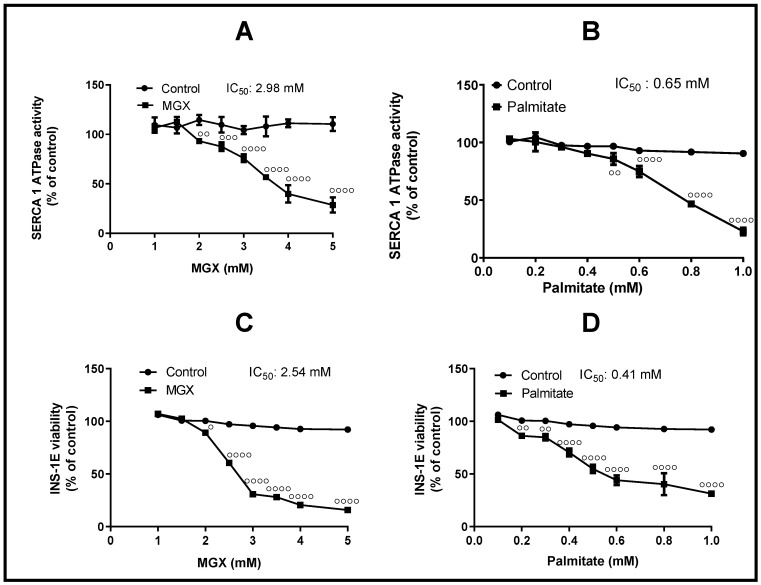
Effect of methylglyoxal (MGX) and palmitate (PAL) on sarco/endoplasmic reticulum Ca^2+^-ATPase (SERCA1a) activity in sarcoplasmic reticulum (SR) and on the viability of INS-1E pancreatic beta cells. SERCA1a activity in SR (**A**) in the absence or presence of increasing concentrations of MGX and (**B**) in the absence or presence of increasing concentrations of PAL. Viability of INS-1E cells (**C**) in the absence or presence of increasing concentrations of MGX and (**D**) in the absence or presence of increasing concentrations of PAL. Individual values represent the averages of three independent measurements, each conducted in at least triplicate. ° *p* < 0.05, °° *p* < 0.01, °°° *p* < 0.001, °°°° *p* < 0.0001 indicate a significant decrease in Ca^2+^-ATPase activity or beta cell viability induced by MGX and PAL compared to non-treated samples.

The inhibitory effects of methylglyoxal (MGX) and palmitate (PAL) on SERCA1a activity and INS-1E beta-cell viability were observed, as depicted in Figure 2. Both compounds exhibited a concentration-dependent reduction in SERCA1a activity, with IC_50_ values of 2.98 mM for MGX and 0.65 mM for PAL (Figure 2A,B). Similarly, Figure 2C,D illustrates the relationship between INS-1E cell viability and increasing concentrations of MGX and PAL, with corresponding IC_50_ values of 2.54 mM and 0.41 mM, respectively.

### 3.3. The Protective Effect of Polyphenols on Methylglyoxal- and Palmitate-Mediated Impairment

**Figure 3:**
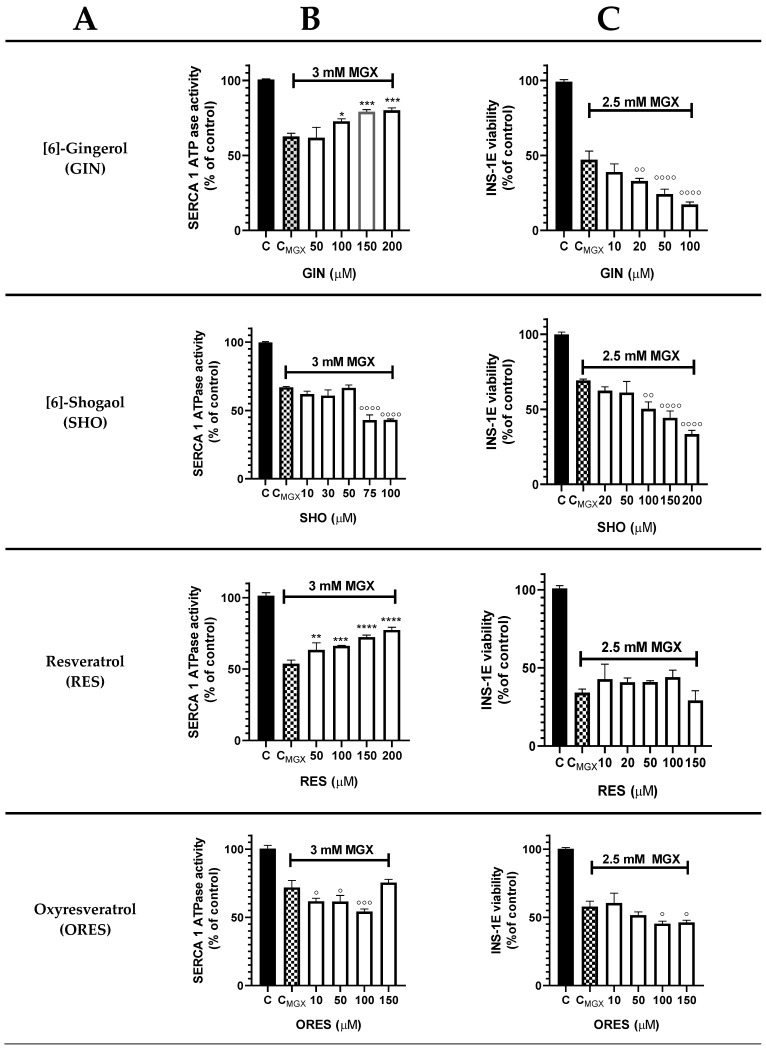

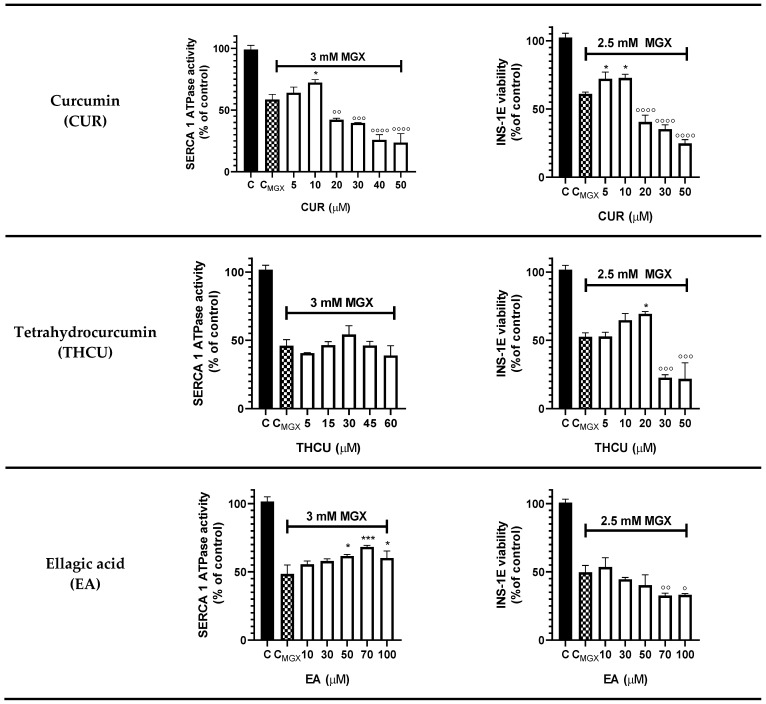
Effect of polyphenols on sarco/endoplasmic reticulum Ca^2+^-ATPase (SERCA1a) activity in the sarcoplasmic reticulum (SR) and viability of INS-1E beta cells in the presence of methylglyoxal (MGX). Polyphenols and their short names (**A**), SERCA1a activity in the SR (**B**) in the presence of 3 mM MGX, and viability of INS-1E cells (**C**) in the presence of 2.5 mM MGX. SR vesicles (1 mg prot./mL) were incubated with polyphenolic compounds (5−200 μM) for 2 min with MGX (3 mM) at 37 °C, pH 7.2. The cells (5 × 10^4^ cells/well) were preincubated for 24 h with polyphenolic compounds (5−200 μM) and MGX (2.5 mM) before the MTT assay. * *p* < 0.05, ** *p* < 0.01, *** *p* < 0.001, **** *p* < 0.0001 denote a significant protection against MGX-induced impairment, while ° *p* < 0.05, °° *p* < 0.01, °°° *p* < 0.001, °°°° *p* < 0.0001 indicate a significant decrease in Ca^2+^-ATPase activity or beta cell viability influenced by individual compounds compared to non-treated samples.

The effects of individual polyphenols on SERCA1a activity and INS-1E beta-cell viability in the presence of IC_50_ values of MGX are depicted in Figure 3. Polyphenols GIN, RES, and EA demonstrated a potent concentration-dependent protective effect against SERCA1a activity decrease induced by MGX. Additionally, CUR (10 µM) prevented MGX-mediated decline in SR Ca^2+^-ATPase activity. On the other hand, compounds SHO, ORES, and CUR (20–50 µM) further decreased SERCA1a activity in the presence of MGX. Regarding INS-1E beta cell viability, only CUR (5 and 10 µM) and THCU (20 µM) exhibited a protective effect against the decrease in viability induced by MGX among the tested polyphenols. The remaining compounds led to an additional viability decrease at increasing concentrations, except for RES, which showed no effect.

**Figure 4:**
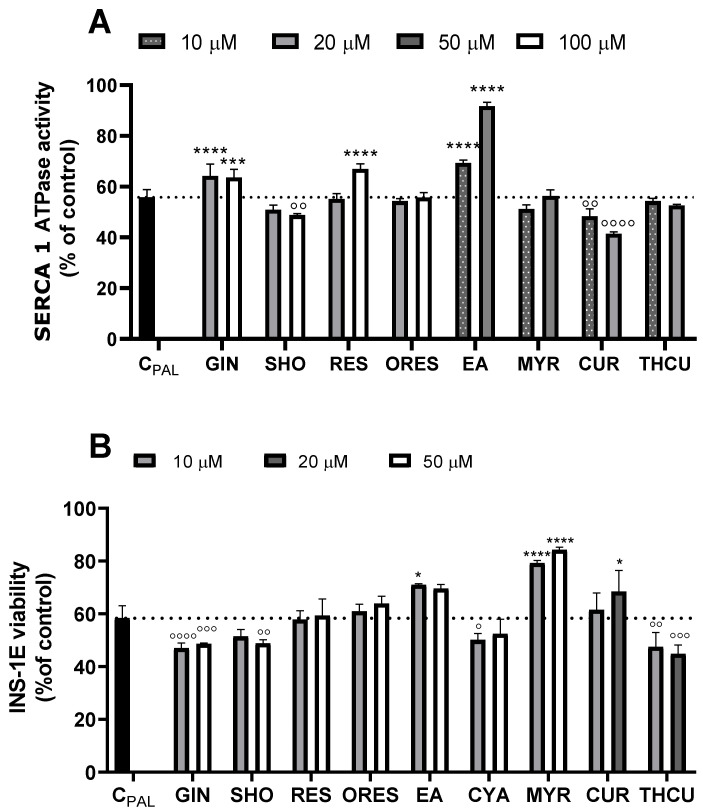
Effect of polyphenols on sarco/endoplasmic reticulum Ca^2+^-ATPase (SERCA1a) activity in the sarcoplasmic reticulum (SR) and viability of INS-1E beta cells in the presence of palmitate (PAL). SERCA1a activity in SR (**A**) in the presence of 0.65 mM PAL and viability of INS-1E cells (**B**) in the presence of 0.4 mM PAL. SR vesicles (1 mg prot./mL) were incubated with polyphenolic compounds (5−200 μM) for 2 min with PAL (0.65 mM) at 37 °C, pH 7.2. The cells (5 × 10^4^ cells/well) were preincubated for 3 h with polyphenolic compounds (5−200 μM) and PAL (0.4 mM) before the MTT assay. * *p* < 0.05, *** *p* < 0.001, **** *p* < 0.0001 denote a significant protection against PAL-induced impairment, while ° *p* < 0.05, °° *p* < 0.01, °°° *p* < 0.001, °°°° *p* < 0.0001 indicate a significant decrease in Ca^2+^-ATPase activity or beta cell viability influenced by individual compounds compared to non-treated samples.

The effects of polyphenols on SERCA1a activity and INS-1E beta cell viability in the presence of IC_50_ values of PAL are shown in Figure 4. EA exhibited the most profound protective effect against PAL-induced SERCA1a activity decrease, followed by GIN and RES. EA (50 µM) alleviated PAL-induced injury almost to control values. Conversely, SHO and CUR evoked an additional decrease in SERCA1a activity in the presence of PAL. Regarding beta cell viability, the highest degree of protection was observed with MYR, followed by EA and CUR. MYR (10 and 50 µM) restored INS-1E viability to approximately 80% of the control cell value.

**Figure 5:**
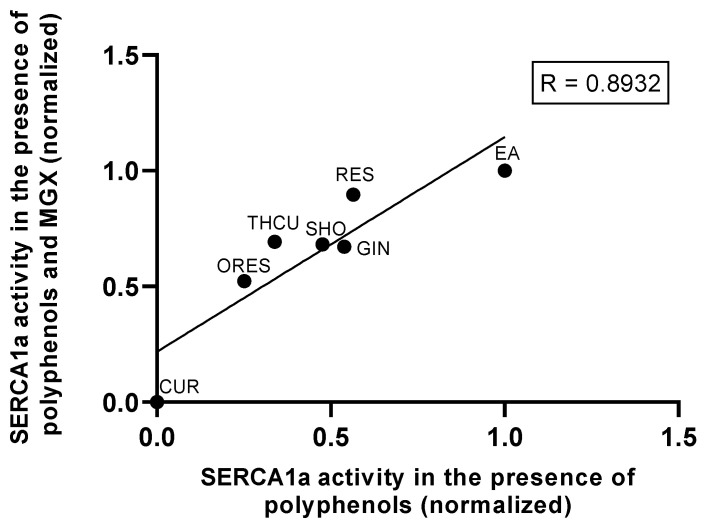
Correlation between sarco/endoplasmic reticulum Ca^2+^-ATPase (SERCA1a) activation by polyphenols and protection against MGX-induced injury. The plot shows the relationship between SERCA1a activity in the presence of 50 µM polyphenolic compounds and its activity in the presence of 3 mM MGX, indicating how polyphenols contribute to the restoration of SERCA1a function under oxidative stress. Values were normalized to control conditions without treatment. The data were rescaled using min–max normalization (0–1).

Our analysis shows a positive correlation between the ability of polyphenols to activate SERCA1a in normal conditions and their ability to protect SERCA1a from MGX-induced impairment (Figure 5). This suggests that polyphenols with stronger modulatory effects on SERCA1a in non-stress conditions retain this activity even when the enzyme is exposed to stressors like MGX, highlighting their potential role in maintaining calcium homeostasis under oxidative stress.

No correlation was found between other parameters, such as SERCA1a activity and beta cell viability or SERCA1a activity/beta cell viability and insulin secretion.

**Figure 6:**
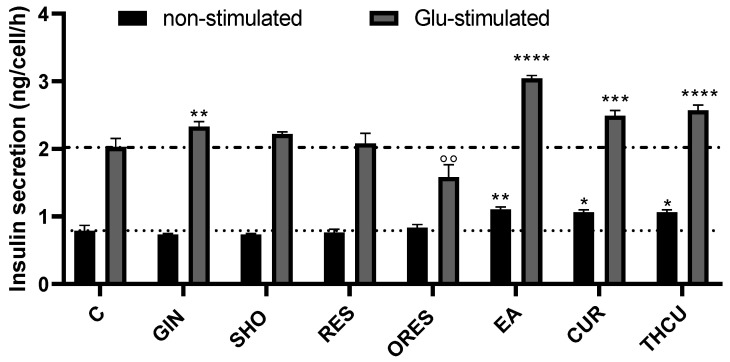
Effect of polyphenols on insulin release from INS-1E beta cells. Cultured cells were pre-treated with polyphenols at a concentration of 50 µM, except for CUR and THCU, which were tested at 10 µM, for 24 h prior to assays following the described methods. * *p* < 0.05, ** *p* < 0.01, *** *p* < 0.001, **** *p* < 0.0001 denote a significant increase, while °° *p* < 0.01 indicates a significant decrease in responses between polyphenol-treated and control cells non-stimulated/post-glucose stimulation.

The effect of the tested polyphenols on insulin release from pancreatic beta cells is summarized in Figure 6. EA, CUR, and THCU exhibited a significant increase in insulin release from both non-stimulated and Glu-stimulated cells compared to the control. GIN elicited a slight increase in released insulin after Glu stimulation but did not induce an increase in non-stimulated beta cells.

Table 1 summarizes the findings, outlining the effects of different polyphenols on SERCA activity and cell viability under control, MGX, and PAL conditions.

### 3.4. In Silico Results

**Figure 7:**
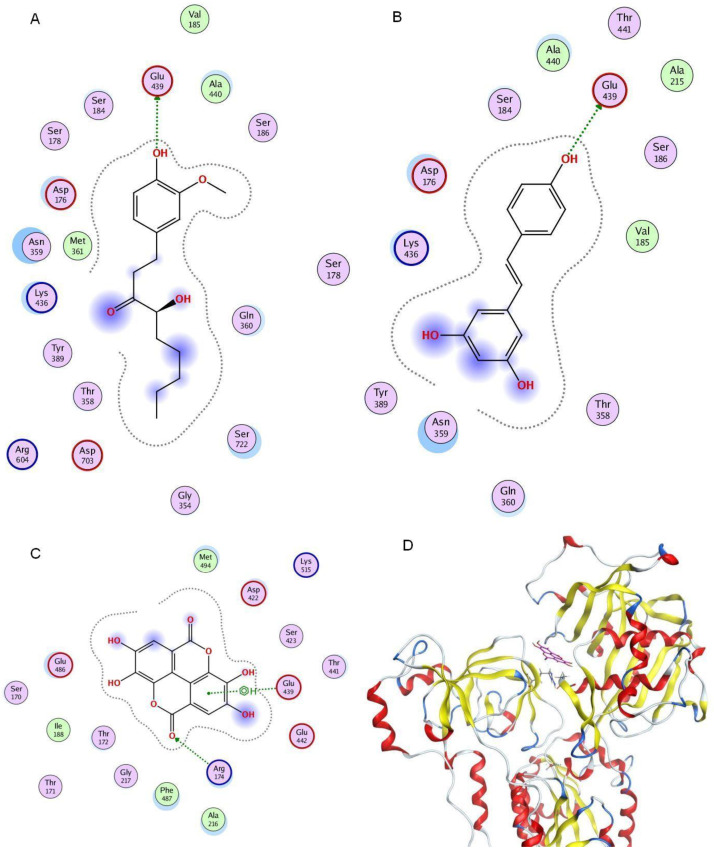
Results from in silico study. (**A**) Position of gingerol bound in sarco/endoplasmic reticulum Ca^2+^-ATPase (SERCA1a) (E2P model, pdb code 2zbe). (**B**) Position of resveratrol bound in SERCA1 (E2P model, pdb code 2zbe). (**C**) Position of ellagic acid bound in SERCA1 (E2P model, pdb code 2zbe). (**D**) Global position of ellagic acid (magenta) bound in the cytoplasmic part of SERCA1 (E2P model, pdb code 2zbe).

Based on the in silico prediction, the compounds GIN, RES, and EA, which increased SERCA1a activity, were observed to bind with Glu439 either via hydrogen bonds (GIN—Figure 7A and RES—Figure 7B) or with an arene–H bond (EA—Figure 7C). Additionally, EA formed an H bond with Arg174 (Figure 7C). Other compounds were found to bind without direct interaction with the Glu439 residue.

**Figure 8:**
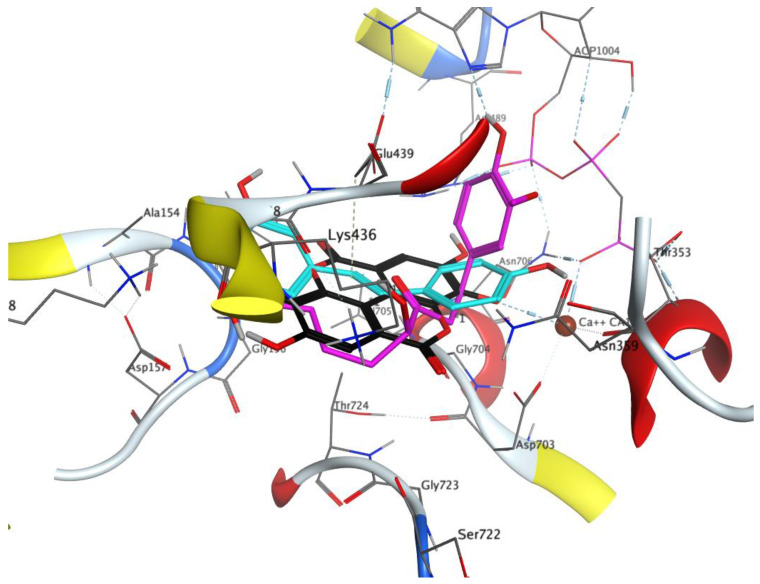
In silico study results showing binding sites for gingerol (magenta), resveratrol (cyan), and ellagic acid (black) in the E1 conformation of sarco/endoplasmic reticulum Ca^2+^-ATPase (SERCA1a) (pdb 4xou).

Docking into the 4xou structure yielded similar placements for all three active compounds (Figure 8). The docking scores for the 4xou structure were comparable to those for the 2zbe structure, suggesting that the binding of these compounds may also occur in the E1 state at the observed concentrations but could persist in the E2 state. The final docking scores were as follows: gingerol: −7.1 (4xou), −6.6 (2zbe); resveratrol: −5.4 (4xou), −6.1 (2zbe); ellagic acid: −7.0 (4xou), −6.0 (2zbe).

## 4. Discussion

The present study explored the effects of various polyphenolic compounds on sarco/endoplasmic reticulum Ca^2+^-ATPase (SERCA1a) activity and pancreatic INS-1E beta cell viability under conditions of oxidative stress induced by methylglyoxal (MGX) and palmitate (PAL). The results demonstrated that certain polyphenols, particularly [6]-gingerol (GIN), resveratrol (RES), and ellagic acid (EA), significantly enhance SERCA1a activity and provide protective effects against the deleterious impacts of MGX and PAL on beta cells.

Elevated levels of PAL and MGX play a role in a range of cellular and molecular alterations associated with diabetes and its complications. Hence, we investigated the impact of these agents on SERCA1a from sarcoplasmic reticulum (SR) within skeletal muscle in vitro as well as within the cellular system of pancreatic INS-1E beta cells to understand the role of SERCA in beta cell regulation. MGX induced a decrease in Ca^2+^-ATPase activity in the SR (SERCA1a) with an IC_50_ of 2.96 mM, while PAL exhibited an IC_50_ of 0.65 mM, indicating its greater efficacy as an inhibitor of SERCA1a. In a model of INS-1E beta cells assessing cell viability, PAL demonstrated a stronger impairment (IC_50_ of 0.41 mM) compared to MGX (IC_50_ of 2.54 mM). The cytotoxicity of MGX and PAL depends on various factors, including the duration of exposure, the concentration, and the specific type of pancreatic beta cell being studied. The mechanism underlying MGX-induced impairment primarily involves interacting with SERCA’s arginine, lysine, or cysteine residues, disrupting its function and leading to dysregulated calcium homeostasis, oxidative stress, and beta cell death [33]. PAL disrupts SERCA activity primarily by depleting ER calcium stores and inducing ER stress. PAL has been shown to alter SERCA2 activity and expression, triggering ER stress via PERK phosphorylation and JNK activation [34]. Moreover, PAL treatment significantly increases the phosphatidylcholine to phosphatidylethanolamine ratio [35] and causes changes in fatty acyl chain length [36], highlighting the critical role of ER membrane composition and thickness in SERCA function. Additionally, excessive levels of MGX and PAL have been shown to increase ROS production, leading to oxidative stress in various cell types, including skeletal muscle and pancreatic beta cells [37,38,39].

Recent findings in experimental diabetic models highlight the significant role of pharmacologic activation of SERCA by allosteric activators, which was associated with the amelioration of ER stress, oxidative stress, ER stress-induced apoptosis, and improvements in specific diabetes-related markers [40,41,42]. Natural products, along with their semi-synthetic derivatives, have constituted the primary source of drugs and remain an important source of chemical compounds for screening and drug discovery. While certain polyphenols have been previously reported to activate specific SERCA isoforms [3], the precise mechanism of their action and their potential protective effects against injury induced by MGX and PAL, particularly in mitigating diabetic complications, remain to be fully elucidated. Our results showed that GIN, RES, EA, and CYA significantly increased SERCA1a activity in sarcoplasmic vesicles, suggesting a potential mechanism for preserving skeletal calcium homeostasis under stress conditions. We hypothesize that the compounds tested directly interact with the crucial amino acids of SERCA1a, leading to changes in SERCA1a activity. While the exact mechanism of SERCA allosteric activation remains unclear, mutation studies suggest a role in the modulatory function of ATP. Clausen et al. [31] found that the Glu439Ala mutation increased the SERCA dephosphorylation rate during the E2P→E2 transition in response to varying ATP concentrations. This suggests that Glu439 interaction with active ligands may enhance SERCA activity, as dephosphorylation is a rate-limiting step in the SERCA1a activity cycle [30]. According to molecular modeling studies, we found that GIN, RES, and EA formed hydrogen bonds with Glu439, suggesting that this interaction may be crucial for SERCA1a activation, indicating a positive correlation between the interaction with Glu439 and the potency to activate SERCA1a.

We found that the concentrations required for efficient SERCA1a activation by natural polyphenols (<30 µM) were significantly higher compared to the synthetic allosteric activator CDN1163 on the SERCA2 isoform (1–10 µM), as shown by Kang et al. [43] and Nguyen et al. [44]. These higher concentrations could increase the risk of aggregation and non-specific interactions, especially at concentrations above 50 µM [45]. However, using DMSO as a solvent may enhance compound stability and solubility [46], ensuring consistent performance under experimental conditions. Our findings suggest that while natural polyphenols have potential as SERCA activators, their higher required concentrations may limit their therapeutic applications compared to synthetic activators like CDN1163.

We observed an inverse relationship between SERCA1a activation by polyphenols and the reduction in beta cell viability (Figure 1). While polyphenols like EA, GIN, and RES enhance SERCA1 activity and thereby improve calcium handling and reduce ER stress, they also display cytotoxic effects at higher concentrations or prolonged exposure, especially documented for CUR [3]. In cell-based assays, concentrations above 50 µM reduced cell viability, likely due to cytotoxicity from the compounds. This aligns with the known biphasic effects of polyphenols, where higher concentrations can cause cellular stress or trigger apoptosis.

The ability of polyphenols to mitigate MGX- and PAL-induced impairment of SERCA1a may involve multiple mechanisms, including competition for binding to SERCA1a key sites and antioxidant effect, attributed to the trapping of reactive carbonyl species. Additionally, in beta cells, other mechanisms involving the regulation of SERCA by sirtuins, PPARs, and PDEs may play a role. Our study identified the polyphenols EA > GIN > RES and MYR > EA ≈ CUR as the most effective compounds in restoring PAL-mediated declines in SERCA1a activity and beta cell viability, respectively. Similarly, the compounds GIN, RES, EA, and CUR preserved SERCA1a activity against MGX-induced injury, and low micromolar concentrations of CUR and THCU (<20 µM) protected against an MGX-induced beta cell viability decrease. These polyphenols are well-recognized for their ability to combat oxidative stress, reduce inflammation, inhibit the aggregation of amyloidogenic peptides, and protect against cellular damage [14], which may be the key mechanisms contributing to their potential therapeutic benefits in conditions associated with oxidative and ER stress, such as diabetes or obesity. Our study showed a positive correlation between the ability of polyphenols to enhance SERCA1a activity and their protective effects against MGX-induced impairment. Specifically, it shows that polyphenols such as EA, GIN, RES, and THCU increased SERCA1a activity both in the presence and absence of MGX, indicating their role in preserving SERCA1a function under oxidative and glycation stress. Interestingly, the most potent SERCA1a-activating polyphenolic compounds failed to prevent MGX- and PAL-mediated impairment in pancreatic beta cells, except EA (10 µM). Our study underscores the lack of direct evidence that SERCA2b activation by polyphenols provides effective protection against MGX- and PAL-induced injury in beta cells. This could be attributed to several factors such as i) the prolonged exposure to cells by polyphenols in cell-based assays (3 h–24 h) versus enzyme assays (2 min), ii) the SERCA2b isoform may not be the primary regulatory mechanism involved in protecting against MGX- and PAL-induced impairment, iii) the compounds may not specifically target the SERCA2b isoform, or iv) the compounds may possess inherent cytotoxic properties, especially at higher concentrations.

Resveratrol (RES) is one of the most extensively studied polyphenols concerning calcium signaling regulation and protecting beta cell function under hyperglycemic conditions. It affects several key pathways, including SIRT, AMPK, mTOR, NF-κB, and TGF-β [3], which are critical for managing oxidative stress, inflammation, energy metabolism, and apoptosis. Our findings show that RES protects SERCA1 activity from the damage induced by both MGX and PAL, with a concentration-dependent effect specifically against MGX (EC_50_ = 111.8 μM). We propose that the protective effect of RES may be linked to its mode of binding to SERCA1. Cheng et al. (2015) suggested RES mitigates pancreatic dysfunction by promoting Nrf2 phosphorylation in an MGX-induced diabetic mouse model [47]. Additionally, RES enhances antioxidant enzyme expression and reduces inflammatory cytokines, thus protecting beta cells from hyperglycemia-induced dysfunction [48]. However, since RES did not prevent the decrease in INS-1E beta cell viability induced by either MGX or PAL in our study, SERCA2b activation does not appear to play a key role in the protective mechanism.

Ellagic acid (EA) demonstrated potent SERCA1a activation (EC_50_ = 33.2 μM), increasing activity by approximately 150% at a concentration of 50 μM, representing the first report of such a finding. Moreover, EA protected SERCA1 activity from the decrease induced by MGX and PAL, with PAL treatment nearly restoring SERCA1a activity to control levels. EA’s potent SERCA1a stimulation and protective effects are likely due to its favorable binding interactions with critical SERCA1a residues and its participation in antioxidant redox reactions, attributed to its multiple hydroxyl groups. These results align with other findings suggesting that EA’s antioxidant and anti-inflammatory properties are particularly potent in combating lipid-induced cellular stress [49]. In pancreatic INS-1E beta cells, EA (50 μM) significantly enhanced insulin release from both glucose-stimulated and non-stimulated beta cells, despite decreasing cell viability, which may suggest an adaptive mechanism. A protective effect of EA under conditions of palmitate-induced oxidative stress was observed, likely associated with its antioxidant properties mediated via the PPARγ signaling pathway [50]. Since PPARγ activation has been linked to restoring SERCA2 levels and protecting beta cells from hyperglycemic stress [51], these findings support EA’s role in preserving beta cell function. Overall evidence indicates that the increased intake of EA is associated with an improvement in obesity and related metabolic complications [43]. Altogether, the protective effects of EA on metabolic diseases may also be linked to its ability to address calcium dysregulation via SERCA activation, suggesting an additional therapeutic approach for improving ER stress-related conditions.

[6]-Gingerol (GIN) induced a concentration-dependent increase in SERCA1 activity (EC_50_ = 36.9 μM). This aligns with previous findings that showed stimulation of SERCA in both skeletal and cardiac muscles, suggesting direct activation of the SERCA protein [52]. According to a recent review of gingerol derivatives, compounds with at least a 6-carbon chain and an o-methoxyphenyl group are crucial for SERCA activity stimulation [53], which may explain why GIN stimulates SERCA1a while SHO does not. GIN concentration-dependently protected against the decrease in SERCA1 activity caused by MGX-induced impairment at 100–200 μM as well as from PAL-mediated injury at 50 and 100 μM, while SHO showed no preventive effect. Since both compounds effectively inhibited AGEs formation by trapping MGX [54], the protective mechanism is more likely associated with the direct interaction of GIN with SERCA1a rather than the antioxidant effect.

The results further revealed that CUR and its metabolite THCU preserved INS-1E beta cell viability in the presence of MGX, with protective effects at low micromolar concentrations and inhibitory effects at higher doses, consistent with hormesis. It has been previously shown that CUR inhibits SERCA1 activity in the skeletal muscle SR by preventing ATP from binding [55]. The protective effects of CUR are consistent with previous findings, which have shown CUR to be a strong antioxidant at low doses, capable of scavenging ROS, reducing lipid peroxidation, and stimulating antioxidant enzymes [56]. CUR’s antioxidant properties, linked to improved glucose and lipid homeostasis in vivo [32], seem to mainly act through the Nrf2 and NF-κB pathways [57]. We also observed that CUR and THCU increased insulin release from both glucose-stimulated and non-stimulated beta cells, indicating not only protection against cellular damage but also the preservation of beta cell function. However, given that excessive antioxidant intake may pose health risks [58], establishing an effective and safe dose is crucial to balance the benefits and potential risks.

The observation that EA, CUR, and THCU promote insulin secretion even under low glucose conditions could raise concerns about the possibility of aberrant insulin release. However, the evidence so far does not explicitly suggest dysregulated exocytosis in the pathological sense. Polyphenols may enhance insulin secretion by modifying insulin sensitivity, optimizing calcium handling, improving beta cell function, and activating non-glucose-dependent signaling pathways, such as PPARγ and AMPK [14,59,60]. Moreover, based on Henquin’s (2021) review translating in vivo to in vitro studies, the threshold for glucose-induced insulin secretion in vitro is approximately 3 mM. This may explain how certain non-glucose stimuli can promote insulin release at this low, yet mildly stimulatory, glucose concentration [61]. Further investigation is needed to determine the exact mechanisms, but these effects point to the broader metabolic impact of polyphenols on beta cell function.

By mitigating the adverse effects of oxidative stress, glucotoxicity, lipotoxicity, and inflammation, polyphenols help preserve and enhance the insulin-secretory capacity of beta cells, which is essential for maintaining glucose homeostasis and preventing the progression of diabetes. In summary, improvements in calcium handling mediated by SERCA activation are crucial for promoting beta cell survival, optimizing insulin secretion, and reducing cellular stress—key factors in sustaining glucose homeostasis. The antioxidant properties of these polyphenols, combined with their interactions with SERCA1a, highlight their therapeutic potential in managing oxidative stress and cellular damage, particularly in diabetes-related complications. The molecular properties and structural features of individual polyphenols could play a pivotal role in their ability to activate and protect SERCA1a from oxidative damage. The findings underscore the potential of targeting ROS generation and calcium dysregulation as a strategy for treating metabolic diseases associated with glucolipotoxicity.

## 5. Conclusions and Future Directions

This research contributes to the growing body of evidence supporting the therapeutic potential of natural compounds in managing conditions associated with impaired calcium homeostasis. This preliminary in vitro study suggests that polyphenols may serve as SERCA1a activators and preserve beta cellular function under conditions of glucolipotoxicity. Specifically, GIN, EA, and RES have shown notable effectiveness in shielding against injury induced by MGX and PAL. The compounds GIN, RES, and EA binding to Glu439 in the SERCA1a P-domain seem critical for enzyme activity stimulation. Future research should aim to elucidate the precise molecular mechanisms by which these polyphenols exert their beneficial effects, including their influence on specific signaling pathways and gene expression, and to validate these findings through in vivo studies.

## Figures and Tables

**Table 1:** Summary of the effects of polyphenols on sarco/endoplasmic reticulum Ca^2+^-ATPase (SERCA1a) activity and cell viability under control, methylglyoxal (MGX), and palmitate (PAL) conditions.

Compound	SERCA1a Activity	INS-1E Viability	MGX		PAL		Insulin Secretion	
			SERCA1a Activity	INS-1E Viability	SERCA1a Activity	INS-1E Viability	Non-Stimulated	Stimulated
**[6]-Gingerol**	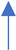	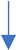	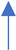	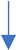	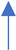	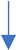	-	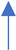
**[6]-Shogaol**	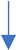	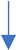	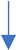	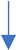	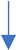	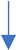	-	-
**Resveratrol**	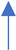	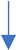	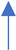	-	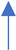	-	-	-
**Oxyresveratrol**	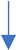	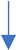	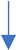	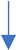	-	-	-	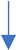
**Curcumin**	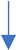	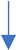	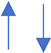	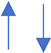	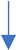	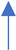	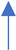	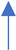
**Tetrahydrocurcumin**	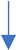	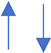	-	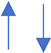	-	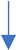	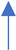	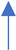
**Ellagic acid**	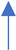	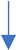	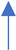	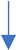	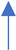	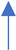	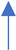	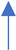
**Cyanidin chloride**	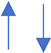	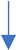	n.d.	n.d.	n.d.	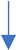	n.d.	n.d.
**Myricetin**	-	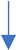	n.d.	n.d.	-	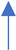	n.d.	n.d.

↓/↑ refers to a decrease/increase in SERCA1a activity, cell viability, or insulin release; “-” represents no significant change; n.d. indicates not determined.

## Data Availability

The raw data supporting the conclusions of this article will be made available by the authors on request.
